# Experiments and Modeling of Three-Dimensionally Printed Sandwich Composite Based on ULTEM 9085

**DOI:** 10.3390/ma17215341

**Published:** 2024-10-31

**Authors:** Radosław Nowak, Dominik Rodak, Stefan Pytel, Przemysław Rumianek, Paweł Wawrzyniak, Daniel Krzysztof Dębski, Agnieszka Dudziak, Jacek Caban

**Affiliations:** 1 Faculty of Automotive and Construction Machinery Engineering, Warsaw University of Technology, 84 Narbutta St., 02-524 Warsaw, Poland; radoslaw.nowak@pw.edu.pl (R.N.); stefan1pytel@gmail.com (S.P.); przemyslaw.rumianek@pw.edu.pl (P.R.); pawel.wawrzyniak@pw.edu.pl (P.W.); daniel.debski@pw.edu.pl (D.K.D.); 2 Faculty of Production Engineering, University of Life Sciences in Lublin, Głeboka 28, 20-612 Lublin, Poland; 3 Faculty of Mechanical Engineering, Lublin University of Technology, Nadbystrzycka 36, 20-618 Lublin, Poland; j.caban@pollub.pl

**Keywords:** layered structures, mechanical testing, analytical modeling, laminate mechanics

## Abstract

This article presents the concept, research, and modeling of a sandwich composite made from ULTEM 9085 and polycarbonate (PC). ULTEM 9085 is relatively expensive compared to polycarbonate, and the composite structure made of these two materials allows for maintaining the physical properties of ULTEM while reducing the overall costs. The composite consisted of outer layers made of ULTEM 9085 and a core made of polycarbonate. Each layer was 3D-printed using the fused filament fabrication (FFF) technology, which enables nearly unlimited design flexibility. The geometry of the test specimens corresponds to the ISO 527-4 standard. Tensile and three-point bending tests were conducted. The structure was modeled in a simplified manner using averaged stiffness values, and with the classical laminate theory (CLT). The models were calibrated through tensile and bending tests on ULTEM and polycarbonate prints. The simulation results were compared with experimental data, demonstrating good accuracy. The 3D-printed ULTEM-PC-ULTEM composite exhibits favorable mechanical properties, making it a promising material for cost-effective engineering applications.

## 1. Introduction

According to the Polish encyclopedia PWN, a composite (lat. compositus—complex, composite) is a material consisting of at least two consciously connected components to receive new and better properties than if they were separated [1]. The materials do not mix with one another and create two main parts of the composite: the matrix, which keeps the structure together and keeps the reinforcement in the right place, and the reinforcement, which, as the name suggests, reinforces the matrix’s mechanical and physical properties and carries all the load [2].

Three main criteria of components’ division can be distinguished—matrix materials, application, and structure—and more subgroups exist within them. Due to this work’s scope, only one of them—the structure category, with structural subdivision—will be covered.

Among these materials, there are subsets of laminates and sandwich composites. The former are layers of material fibers and resin, used as glue, laid on one another with different angles. They are used, for instance, for ski production [3,4]. The latter consists of three layers, where the two outer ones are usually thin, between 0.25 mm and 3 mm, and are responsible for the bending strength and closing of the whole structure [5]. The first inner layer is usually thick and responsible not only for separating the two other layers but also for the compression strength, shear strength, and high Kirchoff’s modulus [6,7]. Cores can take the form of honeycombs, foams, trusses, or simply solid materials, such as the following:

- Polymers;
- Porous polymers based on PU, PVC, and PET;
- Synthetic rubbers;
- Balsa trees, currently replaced with a spherecore [2,3,4].

Additive manufacturing (AM) allows for the creation of sandwich cores in many different and unusual shapes. Geometrically complicated cells that replace well-known honeycombs may perform better in particular circumstances [8]. Another example of AM usage to create a sandwich structure is presented in ref. [9], a paper that describes a new sandwich panel made for the marine industry. The outer layers are made with HDPE filaments and a glass microballoon (GMB), with reinforced HDPE syntactic foam filaments used for the core. The authors proposed different ratios of GMBs in the filament for the cores.

Sandwich composites became popular in the aerospace, marine, and automotive industries due to their high flexural stiffness–weight ratio and increased energy absorption capability [6,7,10,11]. These structures allow for increased stiffness and flexural strength while reducing the costs and/or weight. Certainly, different forms of sandwich composites are characterized by different disadvantages. Very popular honeycombs may absorb the moisture trapped inside the cells, which most likely implicates weight increases and the relocation of the center of gravity. What is more, in case of fire, smoke will be trapped inside, which might be harmful to humans [10]. Foams’ hallmark is their random cellular morphology due to the processes of compression molding, casting, and solid–gas eutectic solidification [12]. Sandwich composites with a core made from solid materials usually inherit their drawbacks. Apart from the components’ disadvantages, the construction itself is also imperfect, with anisotropy and vulnerability to delaminate in the lead. Anisotropic materials are those whose physical properties depend on the direction of examination. Delamination happens when layers of the sandwich composite peel off. Bonding between the layers is a crucial aspect of construction, as damage during experiments or regular use must not be caused by the damage in joints between the layers. Components must be prepared properly to avoid delamination. This means, for example, removing fingerprints and oils, performing wetting improvement, increasing the surface roughness, or carrying out treatment with plasma to increase the surface energy [13]. Moreover, the bonding substance and method of bonding make a significant difference in furthering the endurance of the constructed product.

Parts made by 3D printing are generally anisotropic. Sandwich structures are also not isotropic. The application of the classical laminate theory to model anisotropic materials is one of the possible approaches.

Finding a constitutive model of a 3D-printed part is a complex problem because of its anisotropy and sensitivity in the printing parameters, material, and device quality [14]. For modeling elements produced using 3D printing, the classical laminate theory (CLT) methodology is widely used. In [15,16], each layer of the print is considered as a separate layer for the classical laminate theory. This approach gives excellent accuracy in flexural strength predictions. The authors considered different parameters of printed specimens, such as layer height and raster width. The authors of [17] examined a bi-material composite made of PLA (polylactic acid) and PLA CB (polylactic acid with carbon). The structure was printed on a double extruder 3D printer. The layers were arranged alternately. The specimens were prepared in various combinations of raster angles. CLT was applied to model the structure and gave good results compared to the experiments. The above paper concluded that CLT could be used to predict some mechanical properties, such as the stiffness of 3D-printed structures. An interesting composite modeled with CLT is shown in [18]. Each bead of the filament is enriched with continuous carbon fiber. The authors perform experiments and apply the LAP (commercial software to model laminates based on CLT [19]) to predict results. The LAP results show good agreement with the experiment. A popular way of understating the 3D-printed sandwich is the structure which is all printed from one material, but the outer layers and the core have significantly different shapes. An example of this and modeling proposition is provided by Ghannadpour et al. [20]. The authors decided to use the SLA method to make their structures. They performed a set of basic experiments (tensile test, compressive test, and 3-point bending) for ten different shapes of core cells. Then, it is evaluated with the Finite Element Method.

The following paper is divided into five chapters. Section 1 Chapter one provides information on the motivation behind the chosen topic. Section 2 addresses the printing methods used, the selected materials, and the methodology for tensile and three-point bending tests. It also includes details of the samples used in the experiments. Section 3 describes the modeling approach for the analyzed structures. A simplified model with equivalent stiffness and an approach using the classical laminate theory (CLT) are presented. Section 4 presents and discusses the experimental and simulation results. Section 5 summarizes the entire article and suggests potential directions for future research.

## 2. Materials and Methods

### 2.1. Parameters of 3D Printing

**Figure 1:**
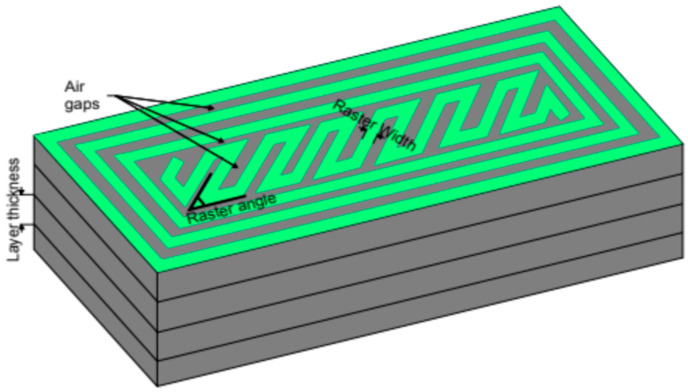
Depiction of air gaps, layer thickness, raster angle, and raster width.

**Figure 2:**
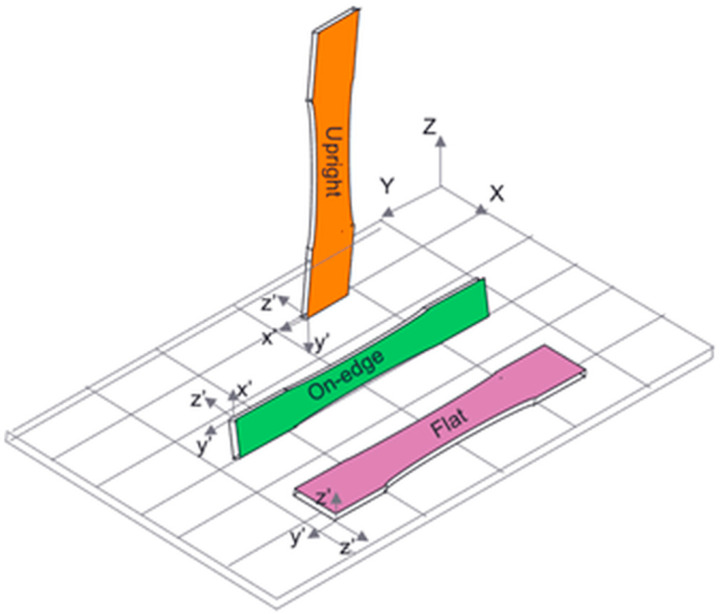
Depiction of various print orientations.

With the rapid growth of material engineering in recent years, AM has joined subtractive methods (cutting, drilling, and grinding). The three-dimensional component, previously modeled in a computer-aided design (CAD) program, can be created by adding the material [21]. Used mainly for prototyping at first, AM is now being adapted also in serial productions, as in 2019, 40% of all online 3D-printed parts were for them [22]. Based on the application, the proper method and materials must be determined. Due to the scope of this work, only Fused Deposition Modeling (FDM) will be covered. It is the most popular method among different AM techniques, accounting for 69% of the total and still gaining popularity. In 2019, 3 times more professionals were using 3D printing than 3 years earlier [22,23]. The principle is straightforward—a material known under the name filament is heated and then becomes molten or semi-molten. It is extruded through the nozzle to be finally deposited on the platform or the top of previously printed layers [8,24]. This method is cheap compared to other techniques, produces less waste, and is relatively fast and easy to perform. Among various advantages, there are some drawbacks with mechanical anisotropy being the most serious one. However, this can be reduced by adequately adjusting the process’s settings. One can change, for example, layer thickness, raster width, raster angle, air gap, temperatures, and many others. Some visualizations can be observed in Figure 1 and Figure 2.

Usually, *X*- and *Y*-axis are parallel to the build platform, while the *Z*-axis is normal to them, along the direction of the part build. Different results may be achieved depending on various materials. Still, most research concludes that the upright product will perform significantly less than those printed on-edge and flat, regardless of the materials used [25]. If the specimen is pulled parallel to the layer deposition direction and applied perpendicularly to its fibers, inter-layer, meaning fusion bond failure, will occur between the layers. Confirmation of this statement can be found, for example, in [26]. The situation is more complex when considering the other two directions. Nevertheless, based on various sources, it was derived that on-edge prints will lead to higher values, especially in flexural strength [26,27], although different results can be observed in the literature [28]. Fibers will withstand most of the applied load if a part is oriented flat or on edge and pulled perpendicular to the layer deposition direction. The error that may appear in such a situation is called trans-layer failure, meaning that a fiber breakage will be observed [26].

The influence of remaining settings is characterized by greater complexity. The materials may differ, but the authors were looking for common patterns that could be applied in their research. The influence of building orientation, raster width, raster angle, air gaps, layer thickness, and their impact on the mechanical properties of a printed part must be described. A raster angle is an angle measured from the *X*-axis to the raster. Raster width refers to the width of the raster pattern used to fill the interior regions of part curves. Air gaps are gaps between two adjacent rasters in the same layer, and layer thickness is simply the thickness of a single layer deposited by the nozzle, which depends on the type of nozzle [29].

Rajch and Siemiński researched the impact of raster angles on flexural strength. Specimens of ABS and three orientations were checked—15 and 105 degrees, 0 and 90 degrees, and 45 and 135 degrees. It needs to be mentioned that all of them were based on cross-raster—fibers in the layer n + 1 were rotated by 90 degrees to the layer n, and the raster width was 0.5084 mm. Moreover, two possibilities were checked for each orientation force parallel to layer deposition and perpendicular to it. The best result, meaning the lowest flexion, was achieved for the fibers with raster angles 45 and 135 degrees, oriented perpendicular to the load. Then, for 15 and 105 degrees, and then for 0 and 90 degrees, both oriented the same way as the first. No air gaps were applied in the samples [30].

Onwubolu and Rayegani conducted research on the influence of raster width, air gaps, and layer thickness on tensile strength. The difference between 45 degrees and 0 degrees was insignificant—a maximum of 5 MPa in favor of 45 degrees. The raster width varied between 0.2032 mm and 0.9652 mm. The growth in strength can be observed within these numbers, along with the growing width. Moreover, the most vital parts were with the negative air gap, which equaled −0.00254 mm. Two possible layer thicknesses were checked—0.127 mm and 0.3302 mm—and it was stated that for the lower thickness, the maximum tensile strength was higher [31].

Gebisa and Lemu researched specimens made of ULTEM 9085 and checked the influence of air gap, raster width, raster angle, contour number, and contour width on tensile strength. These two last ones did not have a significant impact on the results and will not be described. Two possible options of each property were checked—air gap equal to −0.0254 mm and 0 mm; raster width 0.4064 mm and 0.7814 mm; and raster angle 0 and 90 degrees. Both air gap and raster width’s influence were not substantial. A negative air gap was more influential on the specimens with a raster angle of 90 degrees, and low raster width improved tensile strength in all the cases. Raster angle had the most significant impact—specimens with 0 degrees were significantly more robust than those with 90 degrees. The best combination with the highest tensile strength was negative air gap, low raster width, and low raster angle [32,33].

Letcher and Waytashek described the effect of different raster angles on tensile and flexural strength. In the research, all the specimens were made of PLA, and three angles were checked—0, 45, and 90 degrees. The highest tensile strength was achieved at 45 degrees, and then 0 and 90 degrees. On the other hand, the highest flexural strength was in the specimens where the raster angle was 0 degrees (then 45 and 90 degrees) [34].

Letcher, Rankouhi, and Javadpour conducted an experimental study about the correlation between the number of layers, raster angle, and ultimate strength. A total of 204 ABS specimens were tested, and the best results were achieved at 0 degrees, and then 45 and 90 degrees [35].

Sood, Ohdar, and Mahapatra investigated the impact of layer thickness, orientation, raster angle, width, and air gap on tensile and flexural strength. All the specimens were made of ABS and the properties that changed during the research are stored in Table 1. Negative air gaps were not investigated. Conclusions that can be drawn from this experiment are that tensile strength increases with the increase in raster angle, raster width, and air gap. It can also be noted that tensile strength first decreases and then increases as layer thickness increases.

Ahn et al. conducted an experiment in which the influence of air gaps, raster orientation (raster angle), bead width (raster width), color, and model temperature on tensile strength was checked. Only the first three of them will be discussed. The raster angles of 0 or 45 degrees, air gaps of 0 mm or −0.0508 mm, and raster width of either 0.508 mm or 1 mm were examined. It was stated that air gaps and raster angles affect the results significantly, whereas the other properties have a lower impact on them. The highest tensile strength can be achieved with a negative air gap, which equaled −0.0254 mm in a few experiments, but one, in which it was stated that a positive air gap may beneficially influence results. Depending on the material, the raster angle should either be 45 or 0 degrees. Raster width did not significantly influence these results, but it could be stated that the higher it was, the better results were achieved. Lower layer thickness has a positive impact on tensile strength [36]. Although Sood, Ohdar, and Mahapatra reached different conclusions, Shubham, Sikidar, and Chand and Onwubolu and Rayegani confirm these conclusions [31,37]. The highest tensile strength can be achieved by a combination of negative air gap, low raster width, low raster angle, and low thickness.

In terms of flexural strength, the case looks slightly different. Gebisa and Lemu revealed that the low raster width (0.4064 mm) in comparison to the high one (0.7814 mm) and low raster angle (0 degrees) compared to the high one (90 degrees) had a positive impact on the flexural strength of specimens made of ULTEM 9085. In contrast, the air gap did not influence it significantly. If we were to describe the effect of an air gap, 0 mm is better than the negative one (−0.0254 mm) [38]. Wu et al., on the other hand, checked the influence of raster angle and layer thickness in specimens made of PEEK. It was stated that among raster angles 0, 30, and 45 degrees, the highest tensile strength was achieved for the first one (it was cross-raster so 0/90 degrees), and layer thickness equal to 0.3 mm was significantly better than 0.2 mm and 0.4 mm [39]. Motaparti et al. came to the same conclusion about the influence of the raster angle in the research on ULTEM 9085. Moreover, another conclusion was about the negative air gap—the higher, the better flexural yield strength, but it is at the cost of quality. ’However, it should be noted that although the increase in the magnitude of opposing air gap will generally increase the overall strength of the coupon, as the negative air gap rises after a specific value (−0.01905 mm in this case), the surface quality of the fabricated coupon begins to deteriorate’ [27]. In most cases, the best possible output in terms of flexural strength is a low raster angle, negative air gap, high raster width, and medium layer thickness.

Not all researchers came to the same conclusions, and only an average can create general rules, which can be observed for tensile and flexural strength in Table 1.

Different properties, such as the orientation of layers on the bed, the temperature (if possible), speed, width and thickness, filling density, etc., can be adjusted.

Zhang et al. [40] investigated the fracture resistance of specimens made of ULTEM 9085. Based on a linear elastic multilayer fracture mechanics model, they proposed a solution for choosing printing properties to increase the fracture toughness. They observed that delamination along the weak interface gives the best results in fracture toughness. The results may be helpful for predicting and designing fracture-resistant structures.

### 2.2. Applied Materials

Having discussed the characteristics of the process, ULTEM 9085 as one of the materials has to be described. ULTEM 9085, according to its producer Stratasys, is a flame-retardant high-performance thermoplastic for digital manufacturing and rapid prototyping. It is ideal for transportation due to its high strength–weight ratio and FST (flame, smoke, and toxicity) rating [41]. It is necessary to pay attention to postproduction. An additional supporting structure is needed while the FFF technology is applied. That support structures must be removed manually or chemically. Some chemicals can be dangerous for the flammability resistance property of the ULTEM [42]. Contrary to ULTEM 1010, which is pure polyetherimide, ULTEM 9085 is a copolymer of polyetherimide (PEI) and polycarbonate, which can be used in automotive, medical, and aerospace applications [43,44]. However, particular and hardly achievable for amateurs, the 3D print property demands must be met—the extruder temperature 350–390 °C, the print bed temperature between 130 and 160 °C, the heated enclosure, and the printing speed equal to 20–30 mm/s [45]. ULTEM 9085 is very sensitive to the moisture level, and it significantly negatively affects the overall strength of the printed detail [46].

The mechanical properties of the printed detail strongly depend on printing parameters [47,48]. Zadivar et al. [49,50] proved that the tensile strength, failure strain, and Poisson modulus correspond to the print orientation. Tensile strength varies between 46 and 85% of injection molded probes. Depending on printing orientation, the Poisson modulus is measured at level 0.33 (up, *Z*-direction print) to 0.39 (edge, *X*-direction print).

The material’s physical and mechanical properties will be described further. Table 2 shows the comparison with the second material—core (polycarbonate).

Extensive research on other possible cores was carried out. ABS, PLA, PET, and PETG were excluded due to their physical properties or potential problems with bonding. Having chosen the materials, the best printing settings had to be chosen.

The bonding of the materials in the structure was one of the critical elements of the experiment. Surface pretreatments, such as mechanical roughening and plasma activation, were impossible due to significant resource needs. When asked for the best possible solution, the representative of the leading company producing bonding substances that could be used in this research suggested using structural, two-component acrylate resin. On the other hand, Bürenhaus, Moritzer, and Hirsch researched bonding PEI, where they checked one two-component acrylate resin (WELDYX Polyplast), one two-component polyurethane adhesive (DELO-PUR 9694), and four two-component epoxy resins (JOWAT 690.00; LOCTITE M-21 HP; UHU Endfest 300; DELO-AD848). Surprisingly, the best results for two-component epoxy resins-3 reached a lap shear strength of approximately 7.5 MPa, whereas JOWAT 690 was only 5 MPa. In the other cases, adhesion failures could be spotted, which suggests that the two-component acrylate resin may not be sufficient. It was stated that ‘the compatibility between these adhesives and the ULTEM 9085 is poor’. Bearing that in mind, the two-component epoxy resin UHU Endfest 300 was chosen as it was quickly accessible, cheap, and presumably allowed for the avoidance of delamination. The bonding substance’s parameters are presented in Table 3.

The authors decided to create the sandwich composite using the outer layers of ULTEM 9085 and the polycarbonate (PC) core.

The previously presented analysis shows that the best possible output of tensile and flexural strengths could be achieved with the following:

- Negative air gap equal to −0.0254 mm;
- Low raster angle equal to 0°;
- Low raster width equal to 0.4064 mm;
- On-edge orientation;
- Layer thickness 0.3 mm.

Experiments consisted of the 3-point bending test and the tensile test. Both tests were performed on the LaborTech LabTest Z1484 machine (LaborTECH, Oświęcim, Poland). Specimens of pure material were tested to determine material constants like Young’s modulus. The tests were conducted at room temperature using the machine’s dedicated software Test&Motion ver. 1.0 from 2018. Due to the number of specimens, the plots presenting the results were based on the average values. The tests were repeated 3 times to obtain average values.

### 2.3. Preparing Specimens

The whole process of the sandwich composite construction could be described with the presented steps:

- Specimens printing and measurements of the obtained dimensions;
- Grease removal from the specimen’s surfaces;
- Manual bonding and then resting the structures for 24 h.

The testing of the specimens was then to be performed according to standards. The experiment was treated as a preliminary study and an introduction to further exploring the topic. The standards were not strictly followed, and some derogations were introduced. According to [52,53,54], they could be suitable for flexural tests. However, it should not be applied to the specimens that do not break at all or fail on the outer surface of the test specimen within 5.0% [52]. Because Stratasys, the company that produces ULTEM 9085, stated that this material does not break in *XZ* orientation, this norm should not have been applied in this case [41]. Reference [55] could not be used as the four-point method was impossible. The lack of suitable standards led to a choice of [53] as the norm, with few changes introduced. However, the dimensions of the specimens were introduced according to the norm, and the tests were conducted to compare the results of the three types of proposed specimens. After [53]:

- The support span should be equal to 16 (±1) times the depth of the beam;
- Depth should not exceed width;
- The width should be lower than 1⁄4 of the support span;
- Hanging on each side should be at least 10% of the support span but in no case less than 6.2 mm.

**Figure 3:**
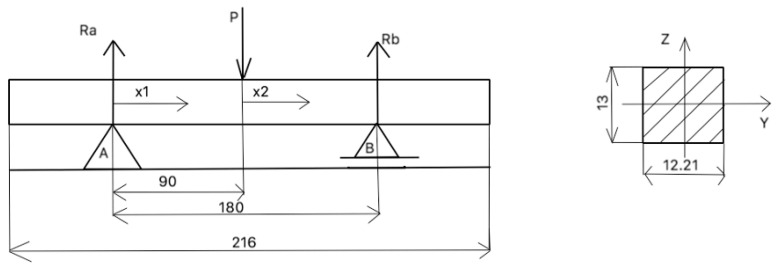
Dimensions of the specimens.

The dimensions had to be adjusted according to the stress appearing during the test. They could not be too low, as the machine could have problems with recording it. For bending tests, cuboids made of pure material were 13.00 high and 12.21 mm deep, the support span was 180.00 mm, and the length was 216.00 mm, as presented in Figure 3.

**Figure 4:**
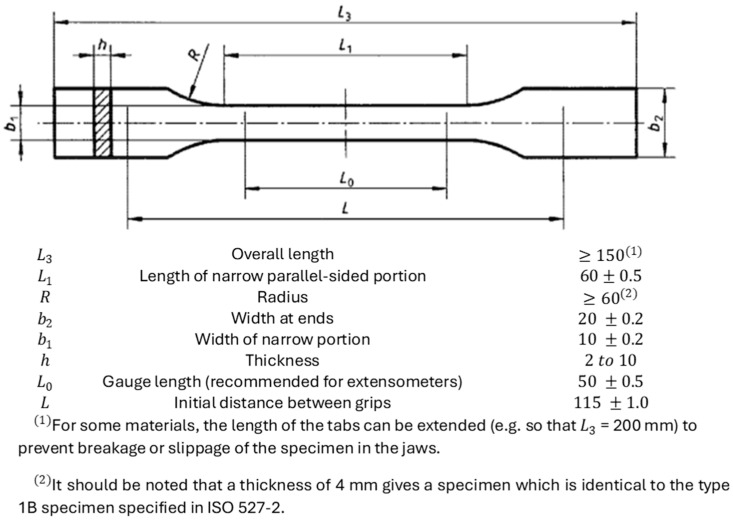
ISO 527-4 guidelines [56].

As the next step, the preparations of the specimens for tensile properties testing had to be made. According to [52,56] should suit composites. However, FDM prints are highly anisotropic, and it is stated in this norm that [56] covers test conditions for isotropic and orthotropic non-unidirectional composites and refers to [57]. Since no other norm would suit this experiment better, the specimens and the machine were prepared according to [56]. Guidelines from the norm can be observed in Figure 4.

Firstly, the printed specimens had to be measured and compared with the desired values. The measurements were carried out using a caliper with an accuracy of 0.02 mm. Table 4 presents the dimensions of the specimens made of ULTEM 9085. Specimens 1, 2, and 3 were cuboids made of pure material for flexural strength testing; specimens 1.1 to 3.2 were outer layers made of ULTEM 9085 meant to be used in sandwich composites in flexural strength testing; specimens 1T, 2T, and 3T were made of pure ULTEM 9085 and meant to be used in tensile strength testing; and specimens 11T to 32T were outer layers made of ULTEM 9085, meant to be used in sandwich composites in tensile strength testing.

In Table 5 the dimensions of the specimens made of polycarbonate are presented. Specimens 4, 5, and 6 were cuboids made of pure PC for flexural strength testing; specimens 4.1 to 4.3 were cores made of PC, meant to be used in sandwich composites in flexural strength testing; specimens 4T, 5T, and 6T were made of pure PC and meant to be used in tensile strength testing; and specimens 4.1T to 4.3T were cores made of PC, meant to be used in sandwich composites in tensile strength testing. It can be easily observed that differences between the obtained and desired dimensions appeared.

**Figure 5:**
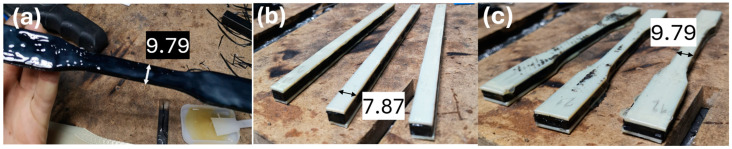
Specimen-making process: (**a**)—applying the glue layer, (**b**)—sandwich beams for bending tests, and (**c**)—composite beams for tensile tests.

Once the prints were ready, sandwich composite construction could be started. Both the cores and outer layers were washed and degreased with isopropanol to create the strongest bond between them. As the company UHU suggested, all the specimens were bonded manually and left for 24 h. Then, the bonded specimens were measured with a caliper once again to check the influence of the bonding substance on the dimensions. The result of it can be observed in Table 6. The process of bonding is presented in Figure 5.

### 2.4. Tensile Test

A tensile strength check was performed as the first test. The extensometer was attached according to the norm to the specimens made of PC, ULTEM 9085, and sandwich composite.

**Figure 6:**
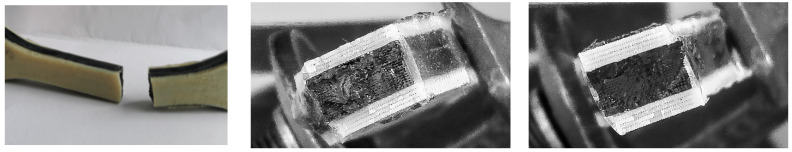
Sandwich composite specimen after the tensile test.

**Figure 7:**
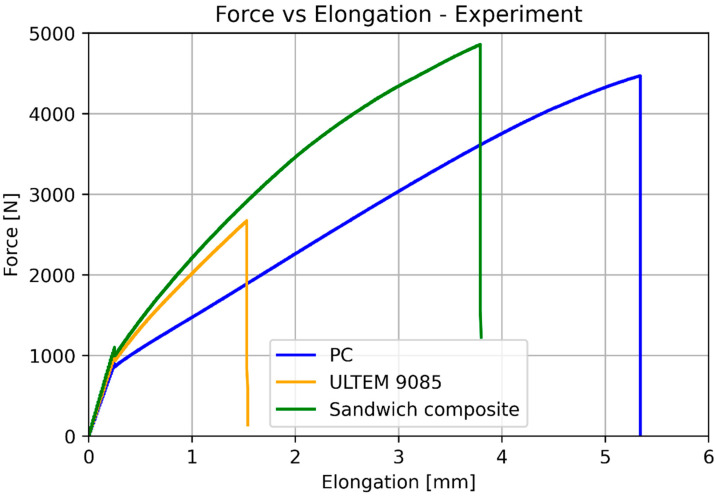
Results of the tensile test for pure ULTEM, PC, and sandwich beam.

**Figure 8:**
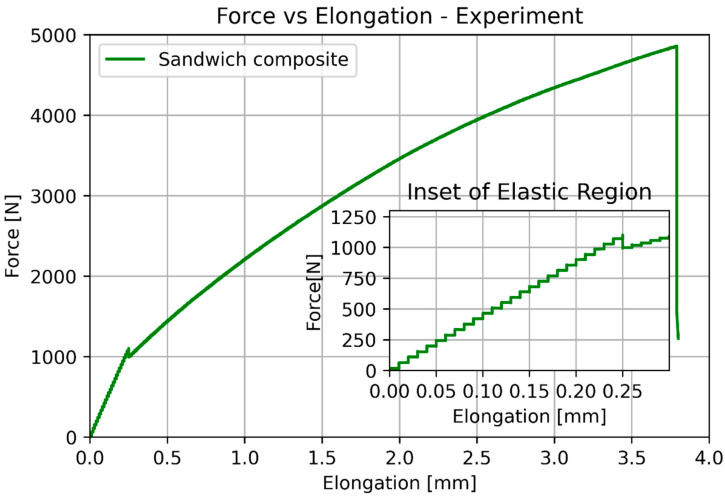
Results of the tensile test for the sandwich beam.

The specimen after the tensile test is presented in Figure 6. The crack appeared in the narrow parallel-sided portion without prior delamination, which was accepted as a satisfactory result. In Figure 6, the cross-section of the specimens was shown, with noticeable smooth perpendicular cracks and layers of the bonded composite parts visible. The average results from testing can be observed in Figure 7 and Figure 8. Due to a high number of points, trendlines had to be drawn. The test was quasi-static, which is why there are ‘steps’ in the plot.

### 2.5. Three-Point Bending

**Figure 9:**
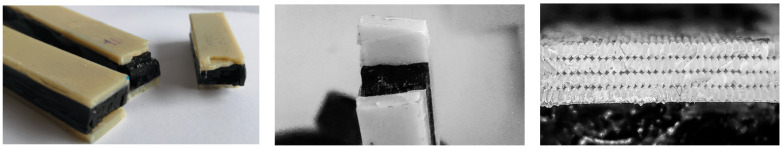
Sandwich composite specimen after the 3-point bending test.

**Figure 10:**
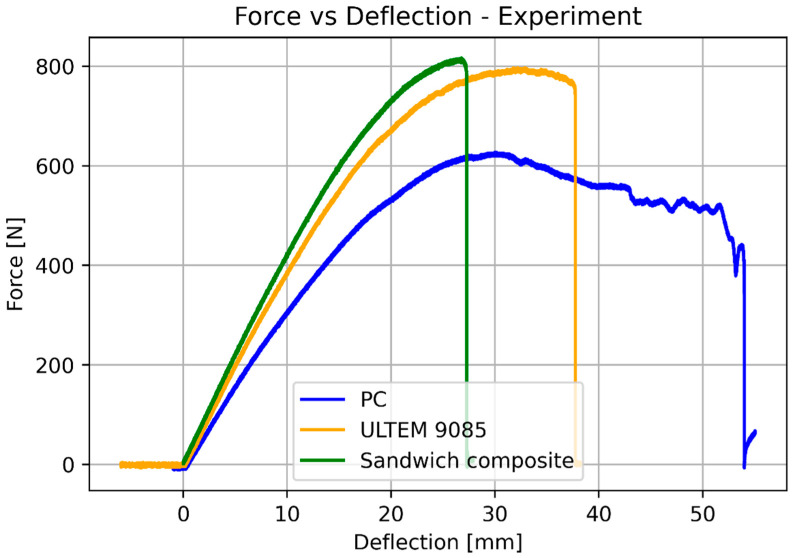
Results of the bending test for pure ULTEM, PC, and sandwich beam.

**Figure 11:**
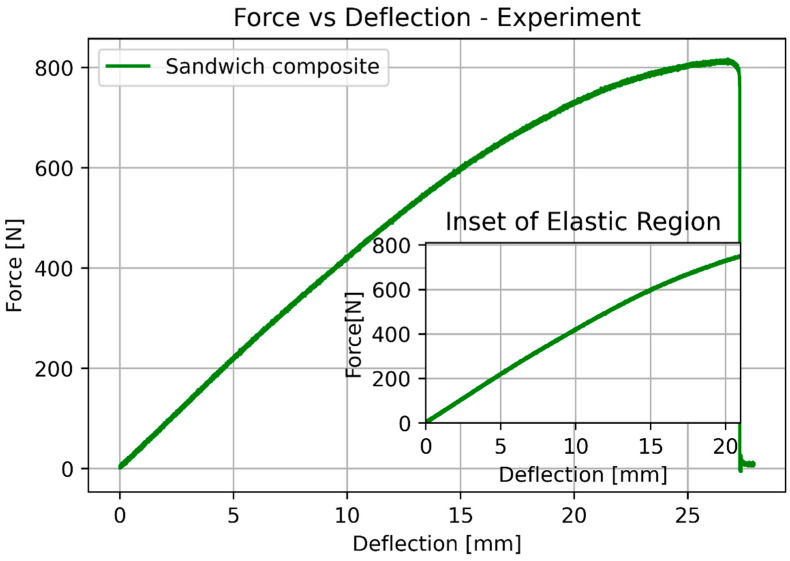
Results of the bending test for the sandwich beam.

The next step was a 3-point bending test. Similarly, as in the previous case, all the specimens were tested and the plots were based on the average values. Figure 9 presents the specimens after the test, and Figure 10 and Figure 11 show the force vs. deflection characteristics.

It can be seen that the crack is perpendicular to the axis of the specimens with a noticeable crack plane in both materials connected with the highest stress and bending moment values. Thus, the whole cross-section of the specimen tears apart simultaneously.

## 3. Calculations

### 3.1. Tensile Calculations

**Figure 12:**
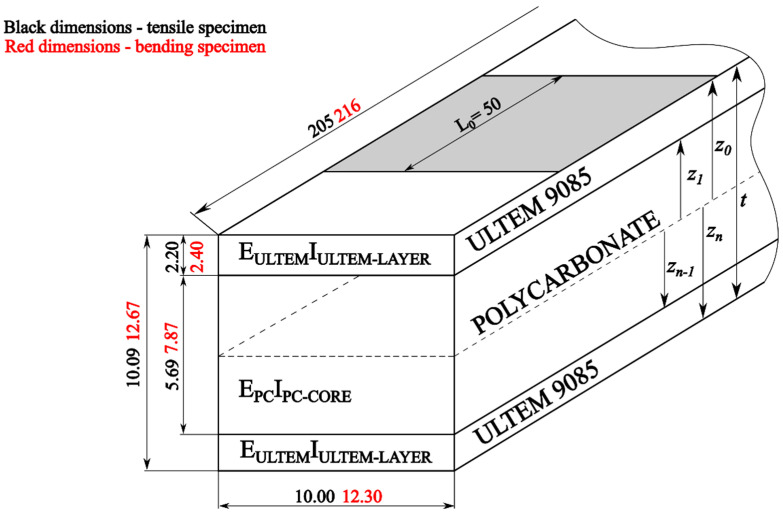
Scheme and dimensions of the sandwich beam.

For the first calculations, Hook’s law (1) was applied to predict the elongation of the specimens. Calculations were conducted for the polycarbonate, the ULTEM, and the sandwich beam, respectively. The cross-section area for each case was determined based on the average dimensions of the printed samples. Exact values are given in Table 4, Table 5 and Table 6. The scheme of the sandwich beam with dimensions is shown in Figure 12.

For the sandwich beam, the resultant stiffness $\tilde{K}$ was determined by Equation (3). The influence of the bonding layer was neglected.

(1) $$F=\frac{\lambda EA}{L_{0}}=\lambda \tilde{K}$$

where

- *F*—force [N];
- λ—elongation [m];
- *L_0_*—gauge length (see Figure 4) [m];
- *E*—Young’s modulus [Pa];
- *A*—cross-section area [m^2^];
- $\tilde{K}$—resultant stiffness.

Thus, the elongation as a function of *F* is equal to the following:

(2) $$\lambda =\frac{FL_{0}}{EA}=\frac{F}{\tilde{K}}$$

The load in the tensile test is applied simultaneously to every layer of the sandwich beam; thus, the resultant stiffness can be represented as a sum of the stiffness of each layer:

(3) $$\tilde{K}=\frac{1}{L_{0}}\left(2\cdot E_{ULTEM}\cdot A_{ULTEM-LAYER}+E_{PC}\cdot A_{PC-CORE}\right)$$

where

- $E_{ULTEM}$—Young’s modulus for ULTEM [Pa];
- $A_{ULTEM-LAYER}$—area of the cross-section of the outer layers made of ULTEM [m^2^];
- $E_{PC}$—Young’s modulus for Polycarbonate [Pa];
- $A_{PC-CORE}$—area of the cross-section of the core made of polycarbonate [m^2^].

**Figure 13:**
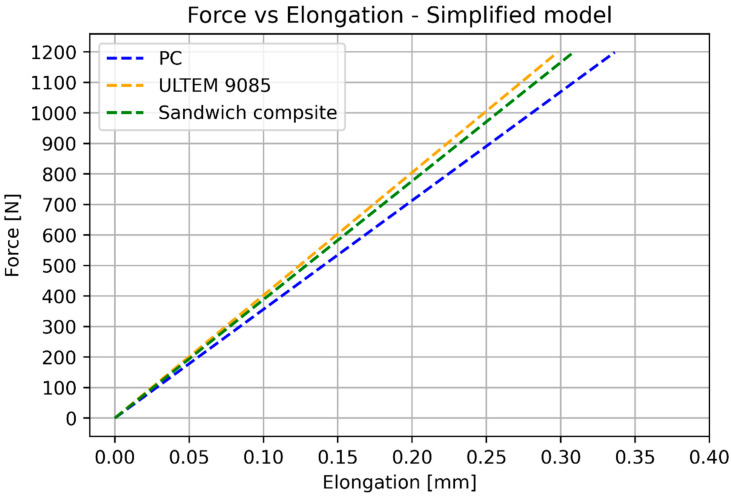
Analytical results for elongation.

The numerical results of the simplified elongation model are shown in Figure 13.

### 3.2. Bending Calculations

**Figure 14:**
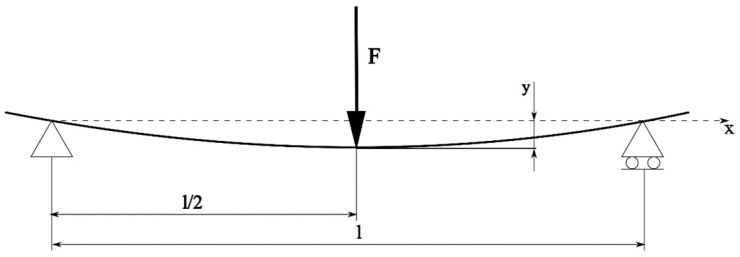
Scheme of 3-point bending.

Analytical deflection calculation in the case of the 3-point bending test was based on the differential equation of the bent axis. In the *xy* coordinate system shown in Figure 14, where axis *x* coincides with the non-deflected beam axis and the *y*-axis is perpendicular and parallel to the load forces, the equation of the bent axis is given as a function:

$$y=f\left(x\right).$$

where $M_{b}$ is bending moment.

Assuming that deflections are relatively small, the radius of curvature is big, neglecting transverse forces; if the EI factor and the cross-section of the beam are constant, it is possible to represent the equation of a deformed beam in Formula (4) [2]:

(4) $$EI\frac{d^{2}y}{dx^{2}}=-M_{b}$$

where $M_{b}$ is bending moment.

After double integration, *y* value is equal to the following:

(5) $$y=\frac{1}{EI}\left[\int \left(\int M_{b}\;dx\right)dx+Cx+D\right]$$

where C and D are integration constants that could be found from boundary conditions. In the case of symmetrical 3-point bending, Equation (5) looks as follows:

(6) $$y\left(x\right)=\frac{1}{EI}\left(\frac{Fl^{2}x}{32}-\frac{Fx^{3}}{12}+\frac{F{\left(x-\frac{l}{2}\right)}^{3}}{6}\right)$$

Thus, the deflection in the middle of the beam is as follows:

(7) $$y\left(x=\frac{l}{2}\right)=\frac{1}{EI}\left(\frac{Fl^{3}}{48}\right)$$

Like the elongation calculations, in the case of the sandwich beam, it is possible to find the resultant flexural stiffness considering both the PC and ULTEM layers.

(8) $$\tilde{EI}=2\cdot E_{ULTEM}\cdot I_{ULTEM-LAYER}+E_{PC}\cdot I_{PC-CORE}$$

**Figure 15:**
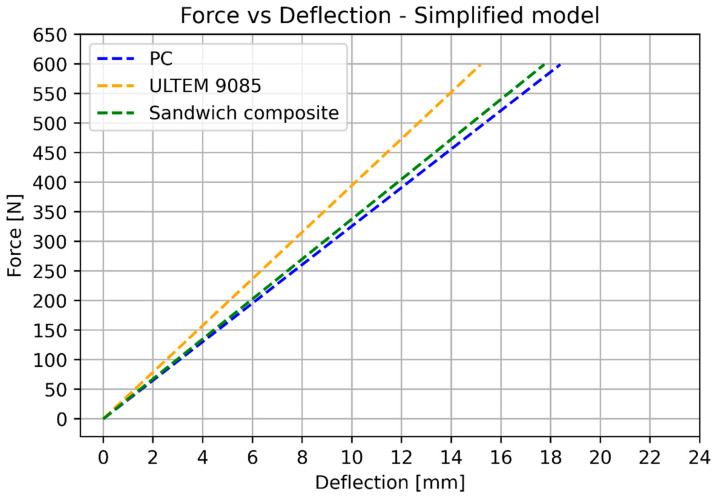
Analytical results for 3-point bending simulations.

The numerical results of the simplified bending model are shown in Figure 15.

### 3.3. Classical Lamination Theory

The sandwich beam made of two materials would be a non-isotropic structure. Simple calculations presented in Section 3.1 and Section 3.2 may be insufficient to reproduce the experimental results accurately. Because of that, the authors decided to model the considered beam with the classical lamination theory (CLT). The CLT is a fundamental theory used to analyze and predict the behavior of laminated composite materials. It provides a simplified approach for determining the mechanical properties and response of laminates consisting of multiple layers of different materials, such as fibers and resins.

The CLT assumes that the individual layers within the laminate are thin. It further believes the layers are bonded perfectly without interfacial defects or delamination. Each layer is considered to be orthotropic. The theory is based on linear elasticity, which assumes that the material’s response to stress is linearly proportional to the applied load. The CLT considers the laminate a combination of individual layers with different orientations and stacking sequences [58].

The sandwich beam is a specific laminate. It consists of three layers with null stacking angles. In the case of the considered beam, each layer is assumed to be isotropic. In Figure 12, the order and dimensions of layers are depicted. The t stands for the total thickness of the laminate, *z* stands for the distance from the neutral axis, and *n* is total number of layers [2].

The constitutive law for the laminate material is given by Equation (9):

(9) $$\left[\begin{array}{c} N_{x}\\ N_{y}\\ N_{xy}\\ M_{x}\\ M_{y}\\ M_{xy} \end{array}\right]=\left[\begin{array}{cccccc} A_{11} & A_{12} & A_{16} & B_{11} & B_{12} & B_{16}\\ A_{12} & A_{22} & A_{26} & B_{12} & B_{22} & B_{26}\\ A_{16} & A_{26} & A_{66} & B_{16} & B_{26} & B_{66}\\ B_{11} & B_{12} & B_{16} & D_{11} & D_{12} & D_{16}\\ B_{12} & B_{22} & B_{26} & D_{12} & D_{22} & D_{26}\\ B_{16} & B_{26} & B_{66} & D_{16} & D_{26} & D_{66} \end{array}\right]\left[\begin{array}{c} \varepsilon_{x}^{0}\\ \varepsilon_{y}^{0}\\ \gamma_{xy}^{0}\\ \kappa_{x}\\ \kappa_{y}\\ \kappa_{xy} \end{array}\right]$$

Thus, layers of the considered beam are oriented parallel to each other:

(10) $$\left[\begin{array}{c} N_{x}\\ N_{y}\\ N_{xy}\\ M_{x}\\ M_{y}\\ M_{xy} \end{array}\right]=\left[\begin{array}{cccccc} A_{11} & A_{12} & 0 & 0 & 0 & 0\\ A_{12} & A_{22} & 0 & 0 & 0 & 0\\ 0 & 0 & A_{66} & 0 & 0 & 0\\ 0 & 0 & 0 & D_{11} & D_{12} & 0\\ 0 & 0 & 0 & D_{12} & D_{22} & 0\\ 0 & 0 & 0 & 0 & 0 & D_{66} \end{array}\right]\left[\begin{array}{c} \varepsilon_{x}^{0}\\ \varepsilon_{y}^{0}\\ \gamma_{xy}^{0}\\ \kappa_{x}\\ \kappa_{y}\\ \kappa_{xy} \end{array}\right]$$

(11) $$\left[\begin{array}{c} \varepsilon_{x}^{0}\\ \varepsilon_{y}^{0}\\ \gamma_{xy}^{0}\\ \kappa_{x}\\ \kappa_{y}\\ \kappa_{xy} \end{array}\right]={\left[\begin{array}{cc} A & B\\ B & D \end{array}\right]}^{-1}\left[\begin{array}{c} N_{x}\\ N_{y}\\ N_{xy}\\ M_{x}\\ M_{y}\\ M_{xy} \end{array}\right]$$

where

- $\varepsilon_{x}^{0},\;\varepsilon_{y}^{0},\gamma_{xy}^{0},\kappa_{x},\;\kappa_{y},\;\mathrm{and}\;\kappa_{xy}$—deformations;
- A, B, and D—block matrices connected to stiffness properties;
- $N_{x},\;N_{y},\;N_{xy},\;M_{x},\;M_{y},\;\mathrm{and}\;M_{xy}$—loads (forces and bending moments).

(12) $$A_{ij}=\overset{n}{\underset{k=1}{\sum }}Q_{ij}^{\left(k\right)}\left(z_{k}-z_{k-1}\right)$$

(13) $$B_{ij}=\frac{1}{2}\overset{n}{\underset{k=1}{\sum }}Q_{ij}^{\left(k\right)}\left(z_{k}^{2}-z_{k-1}^{2}\right)$$

(14) $$D_{ij}=\frac{1}{3}\overset{n}{\underset{k=1}{\sum }}Q_{ij}^{\left(k\right)}\left(z_{k}^{3}-z_{k-1}^{3}\right)$$

The stiffness matrix Q is given by the following (18):

(15) $$Q=\left[\begin{array}{ccc} \frac{E_{1}}{1-\nu_{12}^{2}\frac{E_{2}}{E_{1}}} & \frac{E_{2}\nu_{12}}{1-\nu_{12}^{2}\frac{E_{2}}{E_{1}}} & 0\\ \frac{E_{2}\nu_{12}}{1-\nu_{12}^{2}\frac{E_{2}}{E_{1}}} & \frac{E_{2}}{1-\nu_{12}^{2}\frac{E_{2}}{E_{1}}} & 0\\ 0 & 0 & G_{12} \end{array}\right]\;$$

where

- $E_{1},\;E_{2}$—Young’s modulus in two perpendicular directions [Pa];
- $\nu_{12}$—Poisson ratio [-];
- $G_{12}$—shear modulus [Pa].

Considering Expressions (16) and (17) for the strain and curvature, respectively, it is possible to determine the elongation and deflection of the considered beam.

(16) $$\varepsilon_{x}^{0}=\left[\begin{array}{c} A_{11}^{*}\\ A_{12}^{*}\\ A_{16}^{*}\\ B_{11}^{*}\\ B_{12}^{*}\\ B_{16}^{*} \end{array}\right]N_{x}$$

(17) $$\kappa_{x}^{0}=\left[\begin{array}{c} B_{11}^{*}\\ B_{12}^{*}\\ B_{16}^{*}\\ D_{11}^{*}\\ D_{12}^{*}\\ D_{16}^{*} \end{array}\right]M_{x}$$

Terms with stars are terms of the inverted stiffness matrix. The constitutive model is formulated without width dimension. Because of that, the force and the bending moment are related to the width of the beam. Because of previous assumptions, the elongation and deflection are equal:

(18) $$\frac{d\lambda }{dx}=A_{11}^{*}\frac{N_{x}}{b}\;\lambda =A_{11}^{*}\frac{N_{x}}{b}\cdot L_{0}\;\frac{d^{2}y}{dx^{2}}=-\kappa_{x}=-D_{11}^{*}M_{x},\;M_{x}=\frac{F}{2\cdot b}\cdot x\;M_{x}\left(\frac{l}{2}\right)=\frac{Fl}{4\cdot b}\;y=D_{11}^{*}\cdot \frac{Fl^{3}}{48}$$

where

- b—width of the beam;
- $L_{0}$—gauge length;
- l—length of the beam in case of bending.

**Figure 16:**
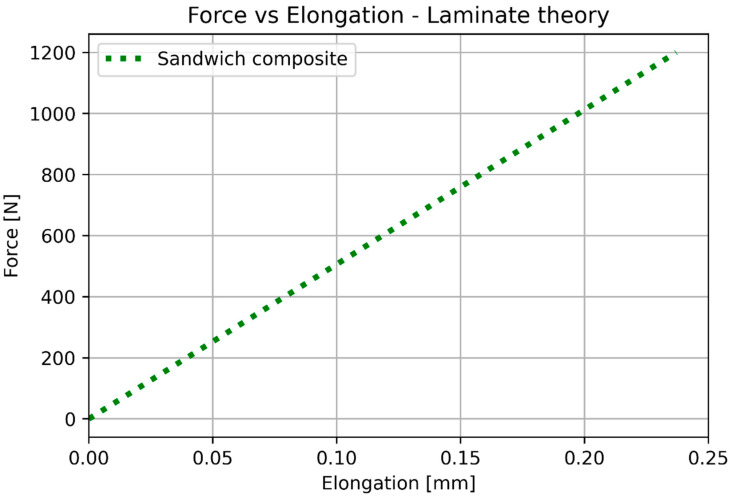
Analytical results for elongation based on the laminate theory.

**Figure 17:**
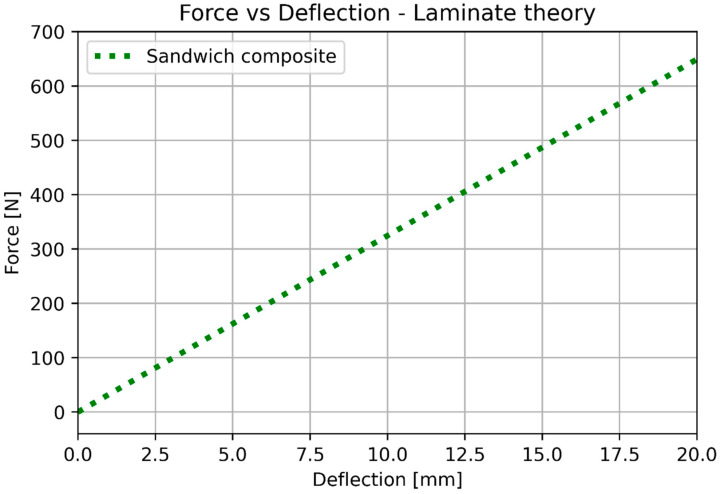
Analytical results for bending based on the laminate theory.

After the substitution of numerical values, Calculations (16)–(18) provide the results shown in Figure 16 (elongation) and Figure 17 (deflection). Table 7 shows the numerical values used for calculating elongation and deflection.

## 4. Results and Discussion

**Figure 18:**
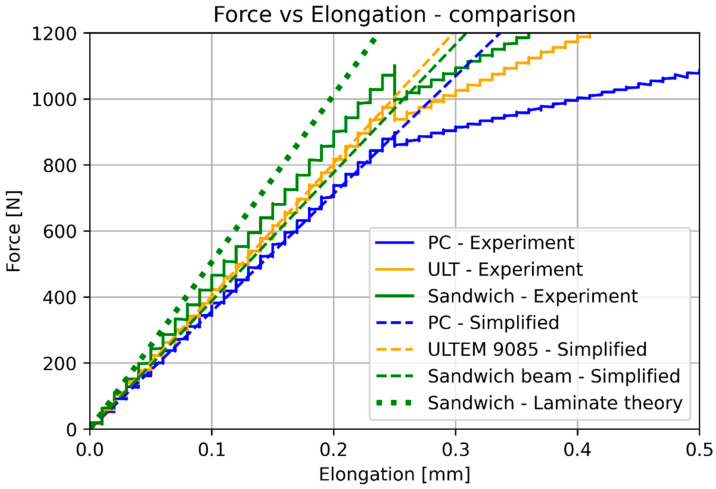
Elongation comparison (linear range).

**Figure 19:**
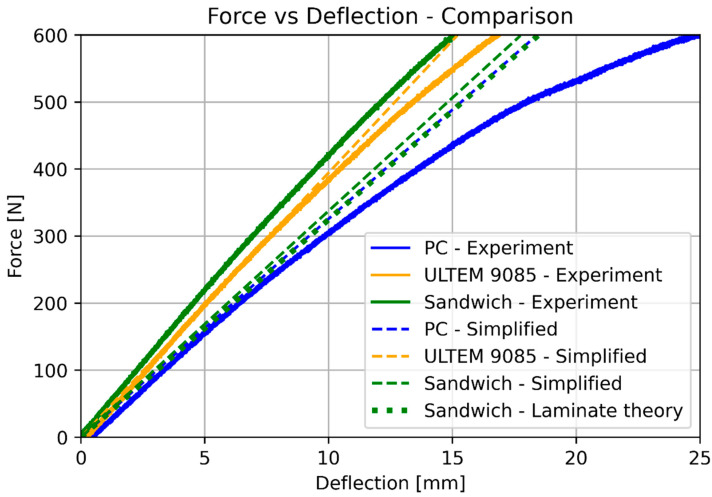
Deflection comparison (linear range).

The basic mechanical properties identified based on experiments and compared to the sheet values for ULTEM 9085, polycarbonate, and sandwich composite are presented in Table 8. Table 9 compares the elongation for the experiment, simplified analysis (Section 3.1), and classical lamination theory (Section 3.3). Table 10 compares the deflection for the experiment, simplified analysis (Section 3.2), and classical lamination theory (Section 3.3). Because of the non-linear behavior of the polycarbonate for small deflections, additional data for 100 N is included. Figure 18 and Figure 19 depict the elongation and deflection comparison, respectively. The axes are limited to a linear range.

In the case of elongation, the classical lamination theory is less accurate (the error is 21.7%) and gives greater stiffness values than the experimental values. The simplified calculation based on Hook’s law directly is more accurate (error 6.73%) and provides lower stiffness values. In the case of elongation, the bonding resin does not significantly influence the results. It is important to remember that the CLT gives an overestimated result.

In the analytical analysis, the results coincided with the ones that had been expected. The sandwich composite’s deflection was higher than the ULTEM 9085 and lower than the polycarbonate. It must be mentioned that the composite’s plot was closer to the PC’s as the PC’s moment of inertia had more influence on the result. The classical lamination theory gives results even closer to the pure PC’s deflection. Both analytical methods underestimate their results in comparison to the experiment. The simplified model based on Hook’s law gives a more accurate result (error 22.63%). The error in the case of the CLT method was 25.58%. The lowest deflection was achieved for the sandwich composite in the experimental analysis. This might have been caused by the bonding substances being pressed into the porous layers of the prints during the bonding process.

During tensile tests, only sandwich composites were destroyed at the assumed point of the specimens (narrow parallel-sided part). The specimens made of the ULTEM 9085 were killed almost in the middle of the radius, whereas the specimens of the PC broke down at the end of the narrow parallel-sided portion. The sandwich composite had different cracks for the outer layers and the core. This may have affected the results of the tests. The shape of the cracks in the PC specimen may lead to the conclusion that the layers did not bond correctly with each other during the printing. Polycarbonate is a rigid material to print in non-professional conditions, as very high temperatures of the bed and extruder are desired.

During the 3-point bending test, the polycarbonate specimens did not break down but remained permanently deformed, whereas the ULTEM 9085 and sandwich composite specimens broke down, with a great amount of energy being released at the same time. The cracks in both were similar, which may indicate that the ULTEM 9085 is a very brittle material.

The simplified way of modeling the elongation and deflection of pure materials is effective, especially in the case of ULTEM 9085, which was printed in professional and stable conditions. The lowest errors in every comparison clearly prove this. The polycarbonate specimen behaved non-linearly, which may be the reason for errors.

Moreover, the results of the analytical analysis should have considered the influence of the bonding resin. On the other hand, its influence would be difficult to describe as it cannot be defined how it penetrated the porous structures of the materials. Bearing in mind the machine’s settings, errors during printing, errors during measurement, caliper’s accuracy, and machine’s accuracy, the differences between the datasheet values and the received ones were not surprising for the authors.

The goals of the preliminary studies on 3D-printed sandwich composite based on ULTEM 9085 were achieved. This means there might be a way to reduce the cost of the parts made of pure ULTEM 9085. On the other hand, this material characterizes extraordinary physical properties, which were not in the scope of this research but should be considered in further studies, especially in terms of bonding it with polycarbonate. As could be observed, resources significantly limited the whole research, and various simplifications had to be made. More surface pretreatments could be introduced, and the experiments could be conducted according to the standards to a greater extent. The next step of the research on creating sandwich composite based on ULTEM 9085 should be printing both ULTEM and PC using a two-nozzle printer. Of course, the influence of the temperatures would have to be considered, but according to the authors’ best knowledge, this is feasible. If the structure created using such a method passes all the necessary tests, then this might be the next step toward the reduction in costs in the production of parts made of ULTEM 9085.

## 5. Conclusions

The article discusses research on composites obtained using 3D printing. The manufacturing technique allows for the obtaining of any shape of a part even after topological optimization. Moreover, 3D printing is a chipless manufacturing method, and there is practically no material loss, except for a small amount used for support.

ULTEM 9085 is used, among others, in the automotive and aerospace industries, where weight reduction and adjusting product requirements to boundary conditions are crucial. ULTEM also has the undeniable advantage of being flame-resistant, which is required in aviation. On the other hand, cost considerations are also considered, as making parts made of pure ULTEM is relatively expensive.

Considering the above, the article presents preliminary studies of samples made from a combination of polycarbonate and ULTEM, thus creating a composite with non-flammable outer layers and a core made of a much cheaper PC.

The subsequent sections of the article present an introduction to the 3D printing method, with particular emphasis on the parameters of the FFF printing method used to produce the samples. The reader is introduced to the analytical methods for calculating the results. The first method utilized average tensile and bending stiffness to calculate the sample’s elongation and deflection, which were determined using the Clebsch method. The second analytical method only concerned calculations related to the composite samples and was derived from the classical laminate theory. It considers Young’s moduli in the longitudinal and transverse directions of the sample, as well as Poisson’s ratio.

In parallel with the analytical calculations, samples were printed from polycarbonate, ULTEM 9085, and a composite of three glued layers.

The samples underwent two types of tests on a universal testing machine: tensile testing along the print fibers and three-point bending, where the cross-section of the loaded beam was subjected to bending and shearing.

The results of the analytical calculations and the experiment are presented as force–elongation relationships for tensile testing and force–deflection relationships for bending. In our opinion, the compiled relationships show similar characteristics to the obtained results, and the values do not significantly differ when comparing the calculations with laboratory tests.

This indicates that the experiment was conducted correctly, though the slight differences are why the team has undertaken further work on testing 3D-printed samples in static and dynamic conditions, which will be presented in future scientific papers.

Using a 3D printer capable of applying successive layers from two different nozzles, a laminate can be obtained without the need for a bonding process.

The research conducted in the article proves that it is possible to obtain a laminate with mechanical properties comparable to elements made entirely of ULTEM 9085. Since the outer layers of the composite will be assumed to be made of ULTEM, the printed samples remain flame-resistant, although a precise determination of this parameter requires further testing.

## Figures and Tables

**Table 1:** Summary of the best properties of the FDM based on various articles.

	Orientation	Raster Angle	Raster Width	Air Gap	Layer Thickness
Tensile strength	On-edge	Low, 0° or 45°, depending on the material	Not much influence, but the higher the better	Negative, −0.0254 mm	The lower, the better
Flexural strength	On-edge	Low, for example, 0° in cross-raster combination (0°/90°)	Not much influence, but the lower the better	Not much influence, −0.0254 mm is commonly used one	Medium

**Table 2:** Mechanical properties of ULTEM 9085 and polycarbonate [47,48].

Material	Young’s Modulus (XZ) [MPa]	Tensile Strength (Ultimate) (XZ) [MPa]	Flexural Strength (XZ) [MPa]	Flexural Modulus (XZ) [MPa]
ULTEM 9085	2150	77	98	2126
PC	1958	42	68	1800

**Table 3:** Specification of UHU Endfest 300 [51].

Chemical Basis	Adhesive Technique	Temperature Range for Use	Combined Tension and Shear Resistance	Mechanical Strength Values	Mixing Ratio
Epoxy resin	Wet adhesion	Between –40 °C and +80 °C	Mixing ratio (by volume) 1:1; testing at room temperature 10 h—500 N/cm^2^ 24 h—1200 N/cm^2^ 5 days—1700 N/cm^2^ 1 month—1700 N/cm^2^	Mixing ratio (by volume) 1:1; testing at room temperature Firm to the touch—6 h Firm enough to use—12 h. Final firmness—24 h	1:1 (other mixing ratios possible)

**Table 4:** Desired and measured dimensions of the specimens made of ULTEM 9085.

Purpose	Specimen No.	Printing Orientation	Desired Dimensions [mm]	Measured Dimension [mm]
Pure material flexural tests	1–3	*XZ*	12.21 × 13.00 × 216.00	Average: 13.20 × 12.30 × 216.00
Composite flexural tests	1.1–3.2	*XZ*	2.44 × 12.21 × 216.00	Average: 2.40 × 12.30 × 216.00
Pure material tensile test	1T–3T	*XZ*	9.75 × 9.90 × 205.00	Average: 9.90 × 10.00 × 205.00
Composite tensile test	11T–32T	*XZ*	2.03 × 9.90 × 205.00	Average: 2.20 × 10.00 × 205.00

**Table 5:** Desired and measured dimensions of the specimens made of polycarbonate.

Purpose	Specimen No.	Printing Orientation	Desired Dimensions [mm]	Measured Dimensions [mm]
Pure material flexural tests	4–6	*XY*	12.21 × 13.20 × 216.00	Average: 12.14 × 12.98 × 216.00
Composite flexural tests	4.1–4.3	*XY*	12.21 × 8.13 × 216.00	Average: 12.10 × 7.87 × 216.00
Pure material tensile test	4T–6T	*XY*	9.90 × 9.75 × 205.00	Average: 10.20 × 9.79 × 205.00
Composite tensile test	4.1T–4.3T	*XY*	9.90 × 5.69 × 205.00	Average: 10.30 × 5.69 × 205.00

**Table 6:** Desired and measured dimensions of the sandwich composites.

Purpose	Specimen No.	Specimen’s Description	Desired Dimensions [mm]	Measured Dimensions [mm]
Beam for tensile test	1A	Core 4.1T with outer layers 11T and 12T	9.90 × 9.75 × 205.00	10.79 × 9.84 × 205.00
	2A	Core 4.2T with outer layers 21T and 22T	9.90 × 9.75 × 205.00	10.98 × 10.04 × 205.00
	3A	Core 4.3T with outer layers 31T and 32T	9.90 × 9.75 × 205.00	10.40 × 9.90 × 205.00
Beam for bending test	1B	Core 4.1 with outer layers 3.1 and 3.2	12.21 × 13.01 × 216.00	12.96 × 13.10 × 216.00
	2B	Core 4.2 with outer layers 1.1 and 1.2	12.21 × 13.01 × 216.00	12.6 × 12.83 × 216.00
	3B	Core 4.3 with outer layers 2.1 and 2.2	12.21 × 13.01 × 216.00	12.86 × 13.00 × 216.00

**Table 7:** Numerical values used for lamination theory calculations.

	Elongation	Deflection
ULTEM 9085 thickness [mm]	2.20	2.40
Polycarbonate thickness [mm]	5.69	7.87
Beam width [mm]	10.3	12.6
Length [mm]	50	180
$\mathrm{ULTEM}\;9085\;E_{1}$ [MPa]	2032.9	2032.9
$\mathrm{ULTEM}\;9085\;E_{2}$ [MPa]	2032.9	2032.9
$\mathrm{ULTEM}\;9085\;G_{12}$ [MPa]	903	903
$\mathrm{ULTEM}\;9085\;\nu_{12}$ [-]	0.3	0.3
$\mathrm{Polycarbonate}\;E_{1}$ [MPa]	1788.86	1788.86
$\mathrm{Polycarbonate}\;E_{2}$ [MPa]	1788.86	1788.86
$\mathrm{Polycarbonate}\;G_{12}$ [MPa]	700	700
$\mathrm{Polycarbonate}\;\nu_{12}$ [-]	0.3	0.3

**Table 8:** Comparison of the datasheet and received values (PC stands for polycarbonate, U for ULTEM 9085, and SC for sandwich composite).

	Young’s Modulus [MPa]			Flexural Strength [MPa]			Tensile Strength [MPa]		
	Sheet Value	Received Value	Error [%]	Sheet Value	Received Value	Error [%]	Sheet Value	Received Value	Error [%]
U	2150	2032.9	5.4	112	100.4	10.4	47	47.21	0.4
PC	1958	1788.86	8.6	89	82.8	6.9	57	52	8.8
SC	-	2114.52	-	-	102.9	-	-	64.62	-

**Table 9:** Elongation in millimeters comparison for 250 N.

Material	Experiment	Simplified Analysis	Error [%]	Laminate Theory	Error [%]
ULTEM 9085	0.07	0.062	12.9	-	-
Polycarbonate	0.07	0.0701195	0.17	-	-
Sandwich Beam	0.06	0.064	6.73	0.0493	21.7

**Table 10:** Deflection in millimeters comparison for 250 N.

Material	Experiment	Simplified Analysis	Error [%]	Laminate Theory	Error [%]
ULTEM 9085	6.05	6.338	4.54	-	-
Polycarbonate * for 100 N	7.54	7.675	1.79	-	-
	* 3.74	* 3.07	* 21.82	-	-
Sandwich Beam	5.73	7.406	22.63	7.700	25.58

* Max load for linear range of deflection.

## Data Availability

The original contributions presented in the study are included in this article; further inquiries can be directed to the corresponding authors.
